# Distemper Outbreak and Its Effect on African Wild Dog Conservation

**DOI:** 10.3201/eid0802.010314

**Published:** 2002-02

**Authors:** Marco W.G. van de Bildt, Thijs Kuiken, Aart M. Visee, Sangito Lema, Tony R. Fitzjohn, Albert D.M.E. Osterhaus

**Affiliations:** * Seal Rehabilitation and Research Centre, Pieterburen, the Netherlands; † Institute of Virology, Erasmus University Rotterdam, Rotterdam, the Netherlands; ‡ The African Wild Dog Foundation, Schiedam, the Netherlands; § Wildlife Preservation Trust Fund, Kilimanjaro Region, Tanzania

**Keywords:** *Morbillivirus*, canine distemper virus, African wild dogs

## Abstract

In December 2000, an infectious disease spread through a captive breeding group of African wild dogs (*Lycaon pictus*) in Tanzania, killing 49 of 52 animals within 2 months. The causative agent was identified as *Canine distemper virus* (CDV) by means of histologic examination, virus isolation, reverse transcriptase-polymerase chain reaction analysis, and nucleotide sequencing. This report emphasizes the importance of adequate protection against infectious diseases for the successful outcome of captive breeding programs of endangered species.

The African wild dog (*Lycaon pictus*) is a highly endangered carnivore found in Africa south of the Sahara. Its population, estimated at <5,500, has declined dramatically in recent decades. Suggested causes for this decline include habitat loss, killing by humans, reduced prey availability, competition with other carnivores, and infectious diseases, including rabies and canine distemper (*1*).

As part of a conservation plan for the African wild dog, a captive breeding program was established in 1995 at Mkomazi Game Reserve, Tanzania, under the auspices of the Government of Tanzania. A founder group of 25 animals was divided into four breeding packs, each housed in a separate fenced enclosure. The founder members and captive-born pups were vaccinated against canine distemper with a vaccine successfully used in seals (*2*), rabies (Rabdomun, Schering-Plough Animal Health, Brussels, Belgium), and parvoviral disease and leptospirosis (combination vaccine: Dohyvac I-LP, Solvay Duphar, Weesp, the Netherlands). The vaccination schedule consisted of three consecutive vaccinations at 2- to 4-week intervals and annual revaccination, most recently in November 1999. Blood samples from a proportion of the vaccinated animals were collected at the time of vaccination to monitor immune response.

## The Outbreak

On December 20, 2000, two of the African wild dogs in one of the breeding packs became ill from an apparently infectious disease. The disease spread rapidly and was first noted in the other breeding packs on January 16, 18, and 22, 2001, respectively. The first deaths occurred on December 21, 2000; deaths peaked from January 30 to February 6, 2001, when 15 of the wild dogs died. The last death was recorded on February 13, 2001. Forty-nine of the 52 animals died during this outbreak.

Neutralizing antibody levels to *Canine distemper virus* (CDV), determined by methods similar to those used in a study of large felids (*3*), were measured in serum samples collected from nine African wild dogs on November 8, 2000. One of the three animals that survived the outbreak had a neutralizing antibody titer of 20; the other two were not tested. The sera from the remaining eight animals had a titer of <20, which is considered to be below the level of protection against canine distemper (*4*).

**Figure 1:**
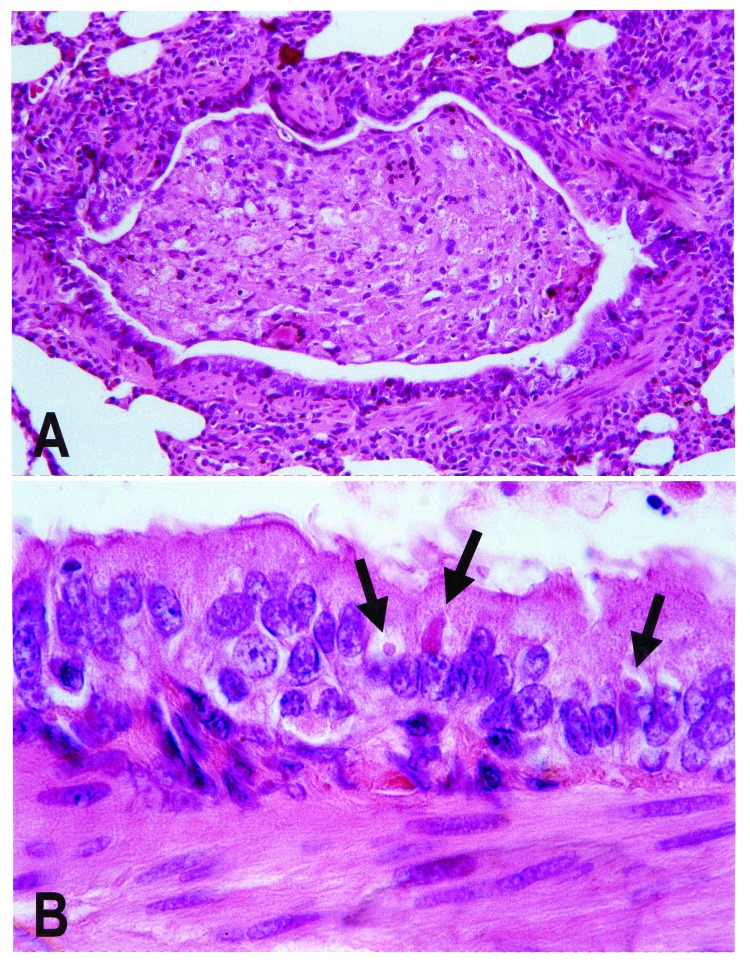
Lung lesions in an African wild dog with canine distemper. Hematoxylin and eosin staining. A. Bronchiole occluded by inflammatory cells and cell debris. B. Detail of A, showing multiple eosinophilic intracytoplasmic viral inclusions (arrows) in bronchiolar epithelium.

**Figure 2:**
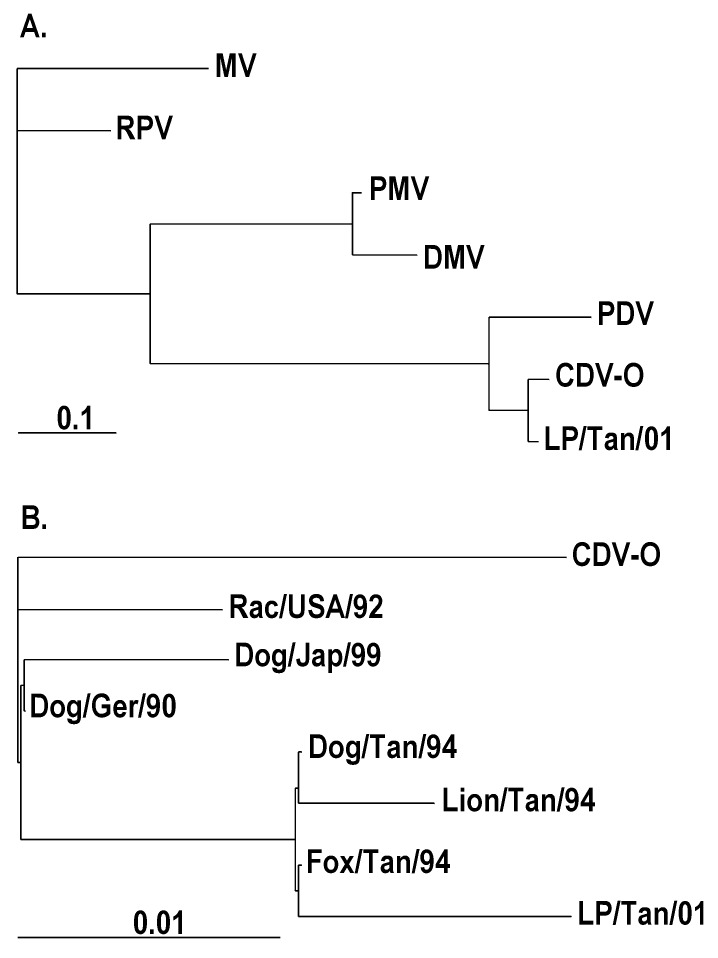
Phylogenetic trees based on a 388-bp *Morbillivirus* P-gene fragment. Maximum likelihood trees were generated by using the SEQBOOT and DNAML program of PHYLIP (Phylogeny Inference Package [*6*]) with 1,000 bootstrap replications. When possible, GenBank numbers of the sequences are given in parentheses. A. Virus from African wild dogs and representative *Morbillivirus* members. MV = *Measles* (Edmonston) *virus*: strain (M89920); RPV = *Rinderpest virus*: RBOK strain (X68311); DMV = dolphin morbillivirus (Z47758); PMV = porpoise morbillivirus (5); PDV = *Phocine distemper virus*-1 (X75960); CDV-O = *Canine distemper virus* (CDV): Onderstepoort strain (AF305419); and LP/Tan/01 = CDV African wild dog, Tanzania (this study). B. Virus from African wild dogs and several CDV strains. CDV-O = CDV: Onderstepoort strain (AF305419); Dog/Ger/90 = CDV from dog, Germany (AF259549); Rac/USA/92 = CDV from raccoon, USA (3); Dog/Jap/99 = CDV from dog, Hamamatsu strain, Japan (AB028915); Dog/Tan/94: CDV from dog, Tanzania (U53715); Fox/Tan/94 = CDV from bat-eared fox, Tanzania (U53714); Lion/Tan/94 = CDV from lion, Tanzania (U53712); and LP/Tan/01 = CDV from African wild dog, Tanzania (this study).

Tissue samples from nine animals that had died were used for histologic examination (n=6), virus isolation (n=2), and reverse transcriptase-polymerase chain reaction (RT-PCR) with *Morbillivirus*-specific primers P1: 5´ATGTTTATGATCACAGCGGT 3´ and P2: 5´ATTGGGTTGCACCACTTGTC 3´, which have been used before for phylogenetic analysis of morbilliviruses (*5*)(n=3). The results of analysis were consistent for all animals tested. The main histologic lesion was broncho-interstitial pneumonia with epithelial necrosis and multinucleated syncytial cells. Eosinophilic intracytoplasmic inclusion bodies, characteristic of canine distemper, were found in the epithelium of lung, kidney, intestine, and urinary bladder (Figure 1). Lung samples scored positive by RT-PCR for a *Morbillivirus* P-gene fragment. Phylogenetic analysis of the nucleotide sequences from the resulting PCR fragments demonstrated that the causative virus was most closely related to CDV (Figure 2A) and clustered with the sequences of CDV strains from domestic dogs (*Canis familiaris*), lions (*Panthera leo*), and bat-eared foxes (*Otocyon megalotis*) from East Africa in the 1990s (Figure 2B). P-gene fragments of the virus isolates from lung samples were identical to the sequences of the PCR products obtained directly from the tissue samples.

## Conclusions

These results show that the primary cause of death of these African wild dogs was CDV infection. Canine distemper is highly infectious for many species of carnivores and causes high death rates in immunologically naïve populations (*7*). It is a known cause of death in free-living African wild dogs (*8*), as well as other wild carnivores, both free-living and captive (*9*,*10*). Based on phylogenetic analysis, the causative virus was a CDV strain circulating in the region in the past decade (*11*,*12*) (Figure 2B).

Potential routes of transmission of this virus to the captive breeding groups are by direct contact with infected domestic dogs or wild carnivores or indirectly by contact with humans or their equipment. Domestic dog populations in some parts of Tanzania are endemically infected with CDV and were considered to be the source of infection for canine distemper in Serengeti lions in 1994 (*13*). Domestic dogs were not present at Mkomazi Game Reserve; however, transmission of CDV from domestic dogs in neighboring villages cannot be ruled out.

Because vaccination with live attenuated virus had been suspected of causing past deaths of African wild dogs (*14*,*15*) the animals in this breeding program were vaccinated with a CDV-ISCOM vaccine, which does not contain live virus. This vaccine protects harbor seals (*Phoca vitulina*) and dogs against phocine distemper virus infection (*2*), which is closely related to CDV, and resulted in protective antibody levels to CDV in African wild dogs monitored at the beginning of this captive breeding program (data not shown). However, the lack of neutralizing antibody titers to CDV in sera of these African wild dogs from November 2000 and the high death rate from canine distemper despite recent vaccination indicate vaccination failure. We are investigating possible reasons for this failure, including problems with application, maintenance of the cold chain, efficacy of the vaccine, and antiviral immune response of the African wild dogs.

Conservation of endangered species, both free-living and captive, has been jeopardized by infectious disease outbreaks in the past (*10*). This outbreak of canine distemper illustrates the disastrous effects that such a disease can have on inadequately protected animals. We therefore conclude that any further attempts to breed African wild dogs in captivity will need to ensure a vaccination regime against canine distemper and other infectious diseases that is both effective in this species and practical to implement under field conditions.
